# Low mean perfusion pressure is a risk factor for progression of acute kidney injury in critically ill patients – A retrospective analysis

**DOI:** 10.1186/s12882-017-0568-8

**Published:** 2017-05-03

**Authors:** Marlies Ostermann, Anna Hall, Siobhan Crichton

**Affiliations:** 1 Department of Critical Care, King’s College London, Guy’s & St Thomas’ NHS Foundation Trust, Westminster Bridge Road SE1 7 EH, London, UK; 2 Department of Critical Care, Guy’s & St Thomas’ NHS Foundation Trust, Westminster Bridge Road, London, SE1 7EH UK; 3 Division of Health and Social Care Research, King’s College London, London, UK

**Keywords:** Acute kidney injury, Central venous pressure, Mean perfusion pressure, Haemodynamics, Risk of progression

## Abstract

**Background:**

The aim was to investigate whether mean perfusion pressure (MPP) calculated as the difference between mean arterial pressure (MAP) and central venous pressure (CVP) was associated with risk of progression from AKI I to AKI III in critically ill patients.

**Methods:**

Retrospective analysis of adult patients admitted to a multi-disciplinary adult intensive care unit (ICU) between July 2007 and June 2009 who developed AKI I and in whom advanced haemodynamic monitoring was initiated within 12 h of diagnosis of AKI I. We compared patients with a MPP above and below the median value in the first 12 h of diagnosis of AKI. Multivariable logistic regression analyses were performed to identify independent risk factors for progression to AKI III, to explore the impact of MAP and CVP separately, and to investigate the impact of MPP in pre-defined sub-groups.

**Results:**

Among 2118 ICU patients, 790 patients (37%) developed AKI I of whom 205 underwent advanced haemodynamic monitoring within 12 h of AKI stage I. Their median MPP was 59 mmHg. AKI I patients with a MPP ≤59 mmHg had a significantly higher risk of progressing to AKI stage III (48.6% versus 34%, respectively; *p* = 0.0034). This association was stronger in patients with ischemic heart disease, congestive cardiac failure or without pre-existing hypertension and in patients with a MAP <65 mmHg for >1 h. As individual components, a raised CVP was independently associated with progression to AKI stage III but MAP alone was not an independent risk factor for AKI progression.

**Conclusion:**

MPP <60 mmHg was independently associated with AKI progression. CVP was the key component of MPP.

## Background

Acute kidney injury (AKI) is one of the most common complications of critical illness affecting 50–60% of patients admitted to the Intensive Care Unit (ICU) [1, 2]. It is associated with serious short- and long term complications, including increased mortality and contributes to significant healthcare costs [3–5]. Worldwide, opportunities are sought to prevent AKI and to reduce the risk of progression.

Haemodynamic regulation of renal blood flow and renal venous pressure are key determinants of renal function. In healthy individuals without systemic hypertension, intrarenal blood flow is auto-regulated at renal perfusion pressures between 60 and 100 mmHg [6]. During critical illness, these processes may be compromised. A raised central venous pressure (CVP) and the resultant increased backward pressure also negatively impacts on renal function, mainly due to renal congestion and increased intra-renal pressure resulting in a fall in glomerular filtration rate (GFR) [7–10]. Most evidence stems from studies in patients with cardiovascular disease where an association between renal venous congestion and the development of AKI has been repeatedly shown [11–16].

To date, there are no established techniques to evaluate and monitor intrarenal blood flow and renal perfusion pressures directly [17]. There are also no reliable means to predict intrarenal haemodynamics from systemic arterial pressures. It has been suggested that mean perfusion pressure (MPP) may serve as a surrogate. MPP is calculated as the difference between systemic mean arterial pressure (MAP) and CVP, ie MPP = MAP – CVP [18] Previous studies have shown that a lower MPP was associated with an increased risk of developing AKI [19]. It remains unknown whether MPP also affects the risk of progression in patients with established AKI.

## Methods

### Aims

The aims of this study were.

i) to explore whether patients with a new diagnosis of AKI stage I and a calculated MPP below the median value had a higher risk of progression to AKI stage III than AKI I patients with a MPP above the median;

ii) to investigate whether MAP or CVP as individual components of MPP have a greater impact on risk of progression from AKI stage I to AKI stage III.

### Setting

Guy’s & St Thomas’ NHS Foundation Hospital is a tertiary care centre with a 43-bed, level 3 multi-disciplinary adult intensive care unit (ICU). The ICU has a fully computerised electronic patient record system where all data are recorded at the time of generation.

### Patient and study design

In this ancillary investigation of a previously reported study [20, 21], we retrospectively analysed a database of all patients admitted to the ICU between July 2007 and June 2009. Using the creatinine criteria of the AKI Network (AKIN) classification, we retrospectively identified patients with AKI stage 1 (AKI I) (i.e., rise in serum creatinine by ≥0.3 mg/dl [≥26.4 μmol/L] or by ≥50% from baseline in ≤48 h) [22]. We only used serum creatinine results obtained during the relevant hospitalization to diagnose AKI in order to comply with the 48-h time window and considered the lowest creatinine result as the baseline value. We selected all patients in whom advanced haemodynamic monitoring had been initiated for clinical reasons within 12 h of the patient meeting the criteria for AKI I.

All patients had an internal jugular vein central venous catheter. Patients with a renal transplant, re-admissions, and patients who left the ICU within 24 h of diagnosis of AKI I or developed AKI stage III within 12 h of diagnosis of AKI I were excluded. The outcome of interest was progression to AKI stage III.

### Data collection

As previously reported [20], we collected demographics, co-morbidities and Sequential Organ Failure Assessment (SOFA) score on admission to ICU and day of AKI I. We also recorded routine haemodynamic parameters obtained by advanced haemodynamic monitoring and arterial lactate concentration during the first 12 h period after diagnosis of AKI I. The MAP and CVP obtained during the first set of advanced haemodynamic monitoring were used to calculate the MPP. Indexed oxygen delivery (DO_2_I) was calculated as DO_2_I = 1.34 X haemoglobin concentration X oxygen saturation X cardiac index. Cumulative fluid balance was determined from all recorded fluid input and output data.

### Statistics

MPP was calculated as the difference between MAP and CVP taken during the immediate 12-h period after diagnosis of AKI I. Characteristics of patients were summarised as median (interquartile range), mean (standard deviation) or frequency (percentage) and compared between patients with a MPP above and below the median value using Mann Whitney, t-test or chi-square tests as appropriate.

The association between MPP and the odds of progressing to AKI III was explored by multivariable logistic regression analysis with adjustment for factors previously shown to be associated with risk of progression, ie. age, indexed oxygen delivery, arterial lactate concentration, cumulative fluid balance and SOFA score [20, 21]. The odds ratios (OR) and respective 95% confidence intervals (CI) were calculated. To explore the possibility of a non-linear relationship between MPP and AKI progression, models were fitted allowing for a quadratic, then cubic relationship between MPP and odds of progression. A further model included MPP categorised into bands of width 10 mmHg. Models were also stratified by presence of pre-existing hypertension, ischaemic heart disease (IHD), congestive cardiac failure (CCF), CVP >15 mmHg or severe hypotension (ie. MAP <65 mmHg for 1 h or more).

The individual impact of CVP and MAP on the effect of MPP was explored using both parameters as continuous measures. The model was adjusted for relevant confounding factors.

Stata 13MP was used to conduct the analyses.

## Results

**Fig. 1:**
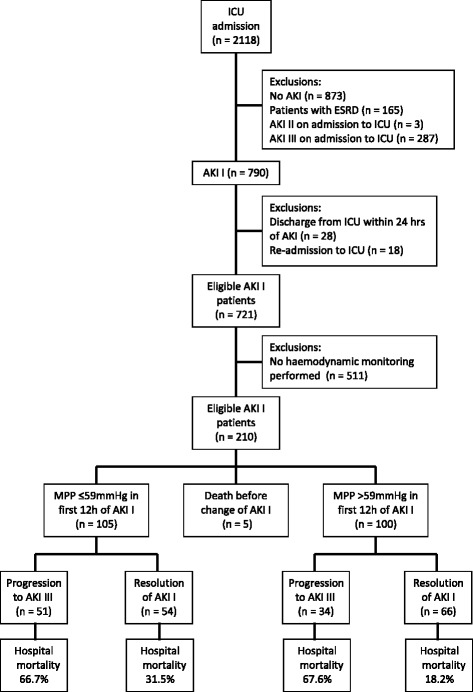
Patient flow. Abbreviations: AKI = acute kidney injury; ESRD = end stage renal disease; ICU = intensive care unit; MPP = mean perfusion pressure

Between July 2007 – June 2009, 2118 patients were admitted to the ICU of whom 790 patients (37%) met the criteria for new onset of AKI I. Sixty-nine patients were excluded. (Fig. 1) Among the remaining 721 patients, haemodynamic monitoring using pulse induced contour or lithium dilution cardiac output technique was initiated by the clinical team in 210 patients within 12 h of AKI I. Five patients were excluded from this analysis as they died before AKI I resolved or progressed. The median age of the remaining 205 patients was 70 years, 67% were male and 44% had underlying IHD or CCF. (Table 1) The median MPP was 59 mmHg.

**Table 1:** Patient characteristics

	Total cohort *n* = 205	MPP ≤59 mmHg *n* = 105	MPP >59 mmHg *n* = 100	*p*-value
Age, median (IQR)	70 (56–77)	71 (56–78)	70 (57–78)	0.66
Male gender, n (%)	138 (67.3)	72 (68.6)	66 (66.0)	0.70
Comorbidities				
IHD / CHF, n (%)	90 (43.9)	45 (42.9)	45(45.0)	0.76
Diabetes, n (%)	38 (18.5)	17 (16.2)	21 (21.0)	0.38
Hypertension, n (%)	79 (38.5)	35 (33.3)	44 (44.0)	0.12
CKD, n (%)	25 (12.2)	12 (11.4)	13 (13.0)	0.73
COPD, n (%)	26 (12.7)	11 (10.5)	15 (15.0)	0.33
CLD, n (%)	11 (5.4)	6 (5.7)	5 (5.0)	0.82
Admission diagnosis				
Post-surgical, n (%)	72 (35.1)	34 (23.3)	38 (38.0)	0.88
Cardiac emergency, n (%)	53 (25.9)	29 (27.6)	24 (24.0)	
Sepsis, n (%)	35 (17.1)	18 (17.1)	17 (17.0)	
Respiratory emergency, n (%)	34 (16.6)	17 (16.2)	17 (17.0)	
Gastrointestinal emergency, n (%)	7 (3.4)	5 (4.8)	2 (2.0)	
Other, n (%)	3 (1.5)	1 (1.0)	2 (2.0)	
Parameters on admission to ICU				
SOFA score, mean (SD)	7.1 (2.8)	7.5 (2.6)	6.8 (2.9)	0.09
APACHE II score, median (IQR)	18 (14–21)	17 (14–21)	18 (13–21)	0.80
Parameters on day of AKI I				
SOFA score, mean (SD)	8.7 (2.7)	9.2 (2.7)	8.1 (2.7)	0.003
Cumulative fluid balance [ml], median (IQR)	2363 (52–3812)	2149 (802–3643)	2568 (994–4116)	0.234
Sepsis, n (%)	122 (59.8)	57 (54.8)	65 (65)	0.138
Parameters within 12 h of diagnosis of AKI I				
DO_2_I [ml/min/m^2^], median (IQR)	362 (277–4836)	347 (270–459)	377 (296–494)	0.132
Arterial lactate [mmol/L], median (IQR)	1.7 (1.3–2.6)	1.8 (1.2–2.6)	1.7 (1.3–2.6)	0.833
MAP <65 mmHg for >1 h, n (%)	107 (52.2)	74 (70.5)	33 (33.0)	<0.001
MAP during 12 h [mmHg], median (IQR)	73 (69–78)	69 (66–73)	78 (73–83)	<0.001
CVP [mmHg], median (IQR)	14 (10–18)	14 (11–18)	11 (9–15)	<0.001
Vasopressor use, n (%)	182 (88.8)	99 (94.3)	83 (83.0)	0.01

*Abbreviations*: *APACHE* Acute Physiology and Chronic Health Evaluation, *AKI* acute kidney injury, *CCF* congestive cardiac failure, *CKD* chronic kidney disease, *COPD* chronic obstructive pulmonary disease, *CLD* chronic liver disease, *CVP* central venous pressure, *DO*_*2*_ *I* oxygen delivery index, *IHD* ischaemic heart disease, *IQR* interquartile range, *MAP* mean arterial pressure, *MPP* mean perfusion pressure, *SD* standard deviation, *SOFA* sequential organ failure assessment

At baseline, there was a significantly larger proportion of patients admitted with acute neurological disorders among those with MPP >59 mmHg. (Table 1) There was no other significant difference between patients with a MPP ≤59 mmHg versus MPP >59 mmHg following AKI I.

On day of AKI I, patients with MPP ≤59 mmHg had a significantly higher SOFA score (mean = 9.2 versus 8.1; *p* = 0.003). (Table 1) Within the initial 12 h period after diagnosis of AKI I, patients with a MPP ≤59 mmHg had a significantly higher CVP (median = 14 versus 11, *p* < 0.001) and required vasopressor support more often (94.3% versus 83%; *p* = 0.010). (Table 1).

### Progression to AKI III

AKI I patients with a MPP ≤59 mmHg during the initial 12 h after diagnosis of AKI I had a significantly higher risk of progression to AKI III compared to AKI I patients with MPP >59 mmHg (48.6% versus 34%, respectively; *p* = 0.0034). Multivariable regression analysis confirmed that SOFA score on day of AKI I and MPP, DO_2_I and first arterial lactate concentration within 12 h of diagnosis of AKI I were independently associated with AKI progression. (Table 2) For each one point increase in MPP, the odds of progression to AKI III decreased by 4.5% (OR = 0.96; 95% CI 0.92–0.996; *p* = 0.031). In sensitivity analyses there was no evidence of a non-linear relationship between MPP and odds of progression.

**Table 2:** Multivariable analysis: Risk factors for progression from AKI I to AKI III

Parameter	OR (95% CI)^a^	*p*-value
First arterial lactate following diagnosis of AKI I [mmol/L]	1.45 (1.12–1.89)	0.005
SOFA score on day of AKI I	1.20 (1.05–1.37)	0.01
First DO_2_I in 12 h period after diagnosis of AKI I [ml/min/m^2^]	0.997 (0.994–0.99)	0.01
First calculated MPP	0.995 (0.92–0.99)	0.03
Age [years]	1.02 (0.997–1.05)	0.09
Cumulative fluid balance on day of AKI I [ml]	1.00 (0.99–1.00)	0.98
MAP <65 mmHg for >1 h in first 12 h after diagnosis of AKI I	0.97 (0.48–1.96)	0.93

*Abbreviations*: *CI* confidence interval, *DO*_*2*_ *I* oxygen delivery index, *MAP* mean arterial pressure, *MPP* mean perfusion pressure, *OR* odds ratio, *SOFA* sequential organ failure assessment

^a^controlled for age, oxygen delivery index, arterial lactate concentration, cumulative fluid balance and SOFA score

### Subgroup analysis

It was hypothesised that the effect of MPP on risk of progression to AKI III may differ in high-risk patients, including those with cardiac disease, pre-existing hypertension, a CVP >15 mmHg or a MAP <65 mmHg for more than 1 h in the 12-h period following diagnosis of AKI I. Subgroup analyses confirmed that MPP was significantly associated with odds of progression to AKI III in patients with IHD or CCF, in patients with MAP <65 mmHg for >1 h and also in patients without pre-existing hypertension. (Table 3) Tests of interaction between each of these variables and MPP were statistically non-significant (*p* > 0.1).

**Table 3:** Subgroup analyses: Adjusted association between MPP and progression to AKI III

Patient cohort	OR (95% CI)^a^	*p*-value
No IHD / CCF	0.98 (0.92–1.04)	0.49
IHD / CCF	0.92 (0.86–0.98)	0.019
MAP not <65 mmHg for >1 h	0.96 0.90–1.02)	0.23
MAP <65 mmHg for >1 h	0.93 (0.87–0.98)	0.013
CVP ≤15 mmHg	0.94 (0.87–1.01)	0.08
CVP >15 mmHg	0.98 (0.92–1.04)	0.47
No pre-existing hypertension	0.93 (0.88–0.99)	0.032
Pre-existing hypertension	0.96 (0.90–1.03)	0.259

*Abbreviations*: *AKI* acute kidney injury, *CCF* congestive cardiac failure, *IHD* ischaemic heart disease, *CVP* central venous pressure, *MAP* mean arterial pressure, *MPP* mean perfusion pressure, *OR* odds ratio, *CI* confidence interval

^a^represents the change in odds of progression to AKI III associated with a one unit increase in MPP adjusted for age, oxygen delivery index, arterial lactate concentration, cumulative fluid balance and SOFA score

### Comparison of impact of CVP and MAP

Multivariable analysis using CVP and MAP as individual components showed that CVP was an independent risk factor for progression to AKI III (OR 1.08; 95% CI 1.02–1.14; *p* = 0.005) after controlling for age, oxygen delivery index, arterial lactate, cumulative fluid balance and SOFA score. In contrast, MAP during the 12 h period after diagnosis of AKI I was not independently associated with risk of progression (OR = 0.96, 95% CI = 0.92–1.00, *p* = 0.079). Further analyses to explore whether the effect of CVP differed by MAP showed that the interaction was statistically not significant (OR = 1.00; 95% CI 0.99–1.01; *p* = 0.88).

## Discussion

This retrospective single-centre study shows that MPP during the 12 h period following diagnosis of AKI stage I is independently associated with risk of progression to AKI III. The association between MPP and progression was particularly strong in patients with IHD or CCF, those without pre-existing hypertension and in patients with a MAP <65 mmHg for >1 h. CVP was the key component of the MPP with independent impact on risk of progression to AKI III whereas MAP was not independently associated with progression.

The finding that elevated CVP can lead to renal dysfunction was first demonstrated in experimental animal studies [8, 23, 24]. Potential mechanisms include transmission of back pressure to the renal veins, increased pressure along the renal vascular tree leading to compression of tubules and decreased net pressure gradient across the glomerulus, ultimately resulting in decreased glomerular filtration.

In humans, the evidence for an association between CVP and AKI stems predominantly from patients with cardiac disease [15]. A sub-analysis of the ESCAPE (Evaluation Study of Congestive Heart Failure and Pulmonary Artery Catheterization Effectiveness) study in patients with decompensated heart failure revealed that among the haemodynamic parameters measured only right atrial pressure correlated with renal function [11]. A different study in 145 patients with acute decompensated heart failure admitted to the Cleveland Clinic confirmed a direct progressive association between baseline CVP and incidence of AKI: when CVP reached >16 or >24 mmHg, the incidence of AKI was 59 or 75%, respectively [12]. Other haemodynamic variables were not independent risk factors for AKI. In cardiac surgery cohorts where systemic venous congestion is a hallmark feature, such as in patients with right valve pathology, AKI is also very prevalent [15]. Williams et al. analysed the data of 1497 patients who underwent coronary artery bypass grafting and had either an ejection fraction <40% or an age >65 years [16]. They showed that for CVP increments of 5 mmHg above the threshold of 9 mmHg, the risk-adjusted odds ratio for AKI was 1.3 (95% CI 1.01–1.65; *p* = 0.045). In congestive heart failure, the increased backward pressure appears to propagate in all districts of the venous system, including renal veins.

We found that the relationship between MPP and AKI progression was primarily based on an independent association between CVP and AKI. In fact, more than 150 years ago, Ludwig and colleagues made similar observations [21]. They showed that if pressure in the renal veins was raised by about 10 mmHg, urine flow was reduced. They attributed this to the histological observation that an increase of venous pressure was associated with distended venules surrounding the distal ends of the tubules resulting in obliteration of the lumen of the tubules. In 1931, using animal models, Winton and colleagues showed that an increase in venous pressure resulted in greater diminution of intrarenal blood flow than a corresponding change in arterial pressure [8]. Our finding that a rise in CVP was independently associated with progression from AKI I to AKI III whereas a fall in MAP in isolation was not an independent risk factor, complements these observations from 100 years ago.

To date, studies have focussed on the link between CVP and risk of AKI. To our best knowledge, our study is the first which analysed patients who had already developed AKI and explored the association between MAP, CVP and risk of progression to severe AKI. Physiologically, our conclusion that a reduced MPP is associated with an increased risk of progression makes sense. The finding that a higher CVP had a greater impact on the effects of progression than a lower MAP is also supported by basic physiology studies in the literature [8, 24].

Measuring renal congestion by imaging is challenging at the bedside and requires extensive patient manipulation, which, in the critically ill patient greatly reduces the practical applicability of any given technique [17]. Based on our data, we suggest that a calculated MPP ≤59 mmHg in the early phase of AKI I may serve as a surrogate marker of increased risk for progression to AKI stage III. Although we did not study any potential interventions, our data also imply that in cardiac patients with AKI stage I and a MPP ≤59 mmHg, further fluid loading may not be advisable if this leads to an increase in CVP. Clearly, more studies are necessary to investigate whether MPP could serve as tool to assess the risk of AKI and a guide for potential therapeutic manipulation, including attempts to reduce CVP.

Our analysis has all limitations of a retrospective single centre study with a heterogeneous patient population. We also acknowledge that we calculated MPP but did not perform invasive renal pressure monitoring or imaging techniques for comparison. Second, we only analysed patients in whom haemodynamic monitoring had been performed for clinical reasons. Third, we defined AKI by serum creatinine results obtained during hospitalisation only. We did not use urine output criteria and may have missed cases with AKI stage I. Finally, the study design was non-interventional, and the association between MPP and progression of AKI does not prove a causal relationship. Whether actively increasing MPP by raising MAP or lowering CVP may reduce the risk of progression to AKI III needs to be evaluated in future studies.

## Conclusions

Our study showed that MPP ≤59 mmHg is independently associated with progression of AKI in ICU and this association is particularly strong in patients with IHD/CCF, those without pre-existing hypertension and in patients with MAP <65 mmHg for >1 h. A raised CVP had a greater impact on AKI progression than MAP.

## Abbreviations

AKI: Acute kidney injury
AKIN: Acute kidney injury network
APACHE: Acute physiology and chronic health evaluation
CCF: Congestive cardiac failure
CI: Confidence interval
CKD: Chronic kidney disease
CLD: Chronic liver disease
COPD: Chronic obstructive pulmonary disease
CVP: Central venous pressure
DO_2_I: Indexed oxygen delivery
ESCAPE: Evaluation study of congestive heart failure and pulmonary artery catheterisation effectiveness
ESRD: End stage renal disease
ICU: Intensive care unit
IHD: Ischaemic heart disease
IQR: Interquartile range
MAP: Mean arterial pressure
MPP: Mean perfusion pressure
OR: Odds ratio
SD: Standard deviation
SOFA: Sequential organ failure assessment
